# Mr. Goodall, on Ol. Terebinth. in Burns & Scalds

**Published:** 1806-10

**Authors:** J. Goodall

**Affiliations:** Epworth


### *To the Editors of the Medical and Phyfical Journal*

GENTLEMEN,

THE favourable reception my case on Hernia met with from you, induces me to send you the following cases ot Burns and Scalds, treated by the stimulant plan, as recommended by Dr. Kentish. The subject of this case was a child, little more than three years old; she was left alone in the room a few minutes, and during her mother's absence began to play with the fire; her clothes immediately came in contact with it, and in the course of a few seconds her mother returned, beholding her little girl all in flames. She was burnt in various parts of the body, nearly from head to foot; her neck, chin, left shoulder, left arm, elbow, both breasts, and one thigh, were dreadfully burnt, the cuticle being entirely removed from those parts; the other thigh, the other arm, and one leg, were slightly burnt.

The child being in great agonies, they applied train oil and water; but as no relief followed that application, her father came over to request I would give him something for his child, who, he said, was very badly burnt. I could not conveniently attend to see the little sufferer on the day the accident happened, but I sent the following liniment, requesting the parts might be well moistened with it every four or five hours. ℞. Spt. terebinth. ℥iv. Ol. lini sine Ign. ℥ij. M.

The next morning, Nov. 13th, I saw her, and was informed she fell asleep about an hour after the liniment was applied, and had a good night; I was also agreeably surprised to find the inflammation nearly removed. I requested the same application might be continued as before, after which I ordered the following ointment. Ung. cera alb. ℥iv. Spt. terebinth. ʒvj. M. to be applied night and morning, the parts to be previously covered with a little hair powder.

I saw her again on the 16th; she had slept well the three preceding nights, and I was astonished to find such rapid improvement in the appearances of the parts; the ointment was continued as before.

23d. The mother called to inform me her child was mending very fast; but the parts became so extremely troublesome from a violent itching, brought on by the healing process, that she got very little rest. I gave her a mixture, a mixture, with the syr. papav. alb. and tinct. opii at bed time, which in some degree removed the irritation on her skin, and procured her better nights.

27th. I saw her for the last time, and was much gratified to find the parts were nearly healed; the cure was completed within the month.

Case II. My own daughter was the subject of this case, and about the same age as the child before mentioned. She was running out of the parlour into the kitchen, and accidentally met the servant, who had a large saucepan in her hand, but luckily it did not contain much boiling water; however, the child was pushed down by the girl, and the contents were thrown upon her. She was scalded in several parts, viz. both breasts, back, neck, shoulder, and one ear, the cuticle being nearly removed from the three latter parts. I happened to be at home at the time, which was a fortunate circumstance for my child; I immediately applied the spirit of turpentine in its pure state, and in a quarter of an hour she became quite easy, and went to sleep. The same kind of ointment was applied, night and morning, as in the preceding Case, and the parts were healed in about three weeks.

I conceive whatever has a tendency to strengthen the opinion of others, in the treatment of any complaints, whether internal or external, but more especially when the treatment has been but recently established or recommended, and if delivered in ever such plain language, provided facts are duly attended to, will be received by the Faculty as deserving to be recorded in the Annals of Medicine. When complaints will not yield to the usual methods, it then becomes the business as well as the duty of every medical man to think for himself; and if by some fortuitous circumstance he hits upon a new medicine, which proves successful in the treatment of any obstinate disease, that medicine of course would be deemed a valuable addition to the Materia Medica, as well as additional cases of its powers would by the evidence of other practitioners. Those, therefore, who have not been fortunate enough in the discovery of any new medicine, are certainly to be commended for having endeavoured to know how far such a method of treatment recommended by others, deserves the approbation of the public.

I am, &c.

